# Modulation of miR-204 Expression during Chondrogenesis

**DOI:** 10.3390/ijms23042130

**Published:** 2022-02-15

**Authors:** Luca Dalle Carbonare, Jessica Bertacco, Arianna Minoia, Mattia Cominacini, Lekhana Bhandary, Rossella Elia, Giovanni Gambaro, Monica Mottes, Maria Teresa Valenti

**Affiliations:** 1 Department of Medicine, University of Verona, 37100 Verona, Italy; luca.dallecarbonare@univr.it (L.D.C.); jessica.bertacco@univr.it (J.B.); arianna.minoia@univr.it (A.M.); mattia.cominacini@univr.it (M.C.); rossella.elia@univr.it (R.E.); giovanni.gambaro@univr.it (G.G.); 2 Department of Neurosciences, Biomedicine and Movement Sciences, University of Verona, 37100 Verona, Italy; monica.mottes@univr.it; 3 Flaskworks, LLC, 38 Wareham St, Boston, MA 02118, USA; lekhana.bhandary@gmail.com

**Keywords:** chondrogenesis, miR-204, mesenchymal stem cells, RUNX2

## Abstract

RUNX2 and SOX9 are two pivotal transcriptional regulators of chondrogenesis. It has been demonstrated that RUNX2 and SOX9 physically interact; RUNX2 transactivation may be inhibited by SOX9. In addition, RUNX2 exerts reciprocal inhibition on SOX9 transactivity. Epigenetic control of gene expression plays a major role in the alternative differentiation fates of stem cells; in particular, it has been reported that SOX9 can promote the expression of miRNA (miR)-204. Our aim was therefore to investigate the miR-204-5p role during chondrogenesis and to identify the relationship between this miR and the transcription factors plus downstream genes involved in chondrogenic commitment and differentiation. To evaluate the role of miR-204 in chondrogenesis, we performed in vitro transfection experiments by using Mesenchymal Stem Cells (MSCs). We also evaluated miR-204-5p expression in zebrafish models (adults and larvae). By silencing miR-204 during the early differentiation phase, we observed the upregulation of SOX9 and chondrogenic related genes compared to controls. In addition, we observed the upregulation of COL1A1 (a RUNX2 downstream gene), whereas RUNX2 expression of RUNX2 was slightly affected compared to controls. However, RUNX2 protein levels increased in miR-204-silenced cells. The positive effects of miR204 silencing on osteogenic differentiation were also observed in the intermediate phase of osteogenic differentiation. On the contrary, chondrocytes’ maturation was considerably affected by miR-204 downregulation. In conclusion, our results suggest that miR-204 negatively regulates the osteochondrogenic commitment of MSCs, while it positively regulates chondrocytes’ maturation.

## 1. Introduction

Mesenchymal stem cells (MSCs) are multipotent adult stem cells traceable in multiple tissues, including umbilical cord, bone marrow and fat tissue [1]. MSCs can self-renew and differentiate into multiple cell types including bone, cartilage, muscle and fat cells [1].

During embryogenesis, cartilage development is initiated by a phase of MSCs’ condensation and chondroprogenitor cells’ differentiation [2]. Chondrogenic differentiation of MSCs is characterized by the upregulation of cartilage-specific genes, such as cartilage oligomeric protein (COMP) and cartilage matrix protein (CMP), and the biosynthesis of extracellular matrix components including collagen type II, IX, aggrecan and fibronectin [3]. Additionally, various factors such as transforming growth factor β (TGFβ), fibroblast growth factor (FGF), bone morphogenetic proteins (BMPs), Wnt and Hegehog signaling pathways are involved in cartilage microenvironment formation [4].

RUNX2 and SOX9 are two pivotal transcriptional regulators in chondrogenesis [5]. RUNX2 is essential for hypertrophic chondrocytes’ maturation, while SOX9 is involved in the differentiation towards articular cartilage formation [6]. It has been demonstrated that RUNX2 and SOX9 physically interact and that SOX9 can inhibit the transactivation of RUNX2 [5]. In addition, RUNX2 exerts a reciprocal inhibition on SOX9 transactivity [5]. RUNX2 is important in the chondroblastic/osteoblastic commitment of MSCs, while SOX9 expression is crucial for the maturation of chondroblasts into mature chondrocytes [7].

Although many previous studies have investigated chondrogenic differentiation, its precise molecular mechanisms still need to be clarified.

Recently, epigenetic factors such as DNA methylation, microRNAs (miRNA) and chromatin structure modifications have been proven to be involved in chondrogenesis or osteogenesis [7,8,9,10]. miRNAs are highly conserved, short noncoding RNAs and may affect both chondroblast differentiation and maturation [8]. In particular, it has been demonstrated that miR-204 shows a tissue-specific profile and that its expression is regulated at both transcriptional and post-transcriptional levels [11]. miR-204 is also involved in different pathological processes by targeting important biological pathways [12,13,14,15]. AKT and p38 as well as BMP2/Runx2/ALP signaling pathways are affected by miR 204 expression during osteogenesis [16,17]. In addition, miR-204 has been reported to be involved in cartilage inflammation, fractures and bone-related diseases [18]. RUNX2 has been identified as being a target of miR-204 [19].

Hence, our aim was to investigate the miR-204 role during chondrogenesis and to identify its relationship with the transcription factors involved in chondrogenic commitment and differentiation.

## 2. Material and Methods

### 2.1. Cell Culture and Chondrogenic Differentiation

Bone marrow-derived human MSCs (hBM-MSCs) were plated into a culture plate flask and cultured in MesenPRO RS Basal Medium for MSCs (12747-010, Gibco, Life Technologies Corporation, 3175 Staley Rd., Grand Island, NE, USA) with a 10% supplement mix and 1% penicillin/streptomycin/amphotericin.

Cultured hBM-MSCs were induced to chondrogenic or osteogenic differentiation using Chondrocyte/Osteocyte Differentiation Basal Medium (A10069-01, Gibco, Life Technologies Corporation, 3175 Staley Rd., Grand Island, NE, USA) according to the protocol supplied by the manufacturer. The medium was changed every 3–4 days after initial plating.

### 2.2. Cell Transfection

Before transfection, MSCs were seeded into 6-well plates or T25 flasks and differentiated to the chondrogenic lineage, as previously reported. At a cell confluency of 60–70%, transfection was carried out according to the manufacturer’s instructions using Lipofectamine 3000 Reagent (L3000-008, Invitrogen by Thermo Fisher Scientific Baltics UAB, Vilnius, Lithuania). MSCs were transfected with anti-miR-204-5p inhibitor (Cat#: AM17000, ID: AM11116; Ambion by Thermo Fisher Scientific) and inhibitor-negative control (Cat#: AM17010, Ambion by Thermo Fisher Scientific, Waltham, MA, USA). The cells were transfected at different time-points during differentiation, i.e., days 3, 7 and 10 of culture. 48 h post transfection, cells were harvested, and the cell pellet was processed for RNA and protein extraction.

### 2.3. Zebrafish

Zebrafish experiments were performed at the CIRSAL (Interdepartmental Centre of Experimental Research Service) of the University of Verona, Italy, under ethical authorization n. 662/2019-PR of 16 September 2019. The embryos were obtained from natural spawning of nacre adults (ZFIN database ID: ZDB-ALT-990423-22), according to standard protocols (27). Zebrafish embryos were grown at 33 °C in water from two days post fecundation (dpf). At the experimental endpoint, zebrafish embryos or adults were euthanized and collected for molecular analyses, as described below. Imaging was performed using a Leica M205FA fluorescence microscope (Leica Microsystems, Wetzlar, Germany).

### 2.4. RNA Extraction and Reverse Transcription

The total RNA and miRNAs were extracted from the cell pellet using the RNAeasy Protect and miRNeasy Quiagen mini kits, which were respectively used, as previously reported [20,21]. The concentration and purity of extracted RNA and miRNAs were measured by a Nanodrop instrument. Then, the RNA and specific miRNAs were reverse-transcribed into cDNA using Applied Biosystems Reverse Transcription kits (4368814 and 4366596, Applied Biosystems by Thermo Fisher Scientific, Baltics UAB, Vilnius, Lithuania), with random hexamers or miRNA-specific primers, according to the manufacturer’s protocol.

### 2.5. Real-Time PCR

PCR amplification was performed in a total volume of 20 µL containing 1^x^ Taqman Universal PCR Master Mix (2X) and 1 µg of cDNA. Taqman gene and miRNA-specific probes were obtained from Applied Biosystems (Thermo Fisher Scientific, Life Technologies Corporation, Carlsbad, CA, USA): (miR-204-5p (000508, 20X, FAM); U6 snRNA (001973, 20X, FAM); Col2a1_m1 (Hs00264051, 20X, FAM); Comp_m1 (Hs00164359, 20X, FAM); Cox2_m1 (Hs00153133, 20X, FAM); Runx2_m1 (Hs01047973, 20X, FAM); Sox9-m1 (Hs00165814, 20X, FAM; β-actin (4326315E 20X VIC)). Real-time RT-PCR was carried out both in multiplex and singleplex using U6 and β-actin as internal references. Thermocycling and signal detection were performed with the QuantStudio 3 Real-Time PCR system. 2^−∆Ct^ was used to detect the relative expression of target genes and miRNAs. The PCR stage was repeated for 40 cycles. The data were analyzed by QuantStudioDesign & Analysis Software. In particular, at least three independent analyses were performed, each analysis was performed in triplicate, and C*_t_* values were averaged. Because C*_t_* values vary linearly with the logarithm of the amount of RNA, this average represents a geometric mean.

### 2.6. Protein Extraction and Western Blot

Protein lysates were collected, and the total protein concentration was determined using a BCA protein assay, as previously reported [6]. Protein samples were prepared by adding 1^x^ Laemmli’s sample buffer with 2-mercaptoethanol, heated for 5 min at 99 °C and separated by SDS-PAGE electrophoresis, followed by transfer onto a PVDF membrane. Precision Plus Protein Standards (161-0376, Bio-Rad Laboratories, Hercules, CA, USA) or PageRuler™ Plus Prestained Protein (PI26620, Thermo-Fisher Scientific) were used. Then, the primary (RUNX2 (8486) Cell Signaling Technology, Danvers, MA, USA; beta-Catenin (PA5-19469), Thermo Fisher Scientific; Aggrecan (MA3-16888), Invitrogen by Thermo Fisher Scientific; Thermo Fisher Scientific; β-actin (BA3R), Thermo Scientific; ERK (137F5), Cell Signaling; p-ERK (D13.14.4E), Cells Signaling) and secondary (Anti-mouse (7076) Cell Signaling; Anti-rabbit (7074) Cell Signaling) antibodies were incubated with PVDF membranes, and chemiluminescent signal acquisitions were obtained with the Uvitec Imager. ImageQuant software (GE Healthcare, Little Chalfont, UK) was used to perform densitometric analyses, as previously reported [22].

### 2.7. Statistical Analysis

The results were expressed as the mean ± SD. The statistical analysis was assessed by a Student’s paired-test. Differences were considered significant with *p* < 0.05. Analyses were carried out in two or three independent analyses in triplicate. Statistical analyses were performed using SPSS for Windows, version 22.0 (SPSS Inc., Chicago, IL, USA).

## 3. Results

### 3.1. miR-204-5p Expression Increases during Chondrogenic Differentiation

**Figure 1:**
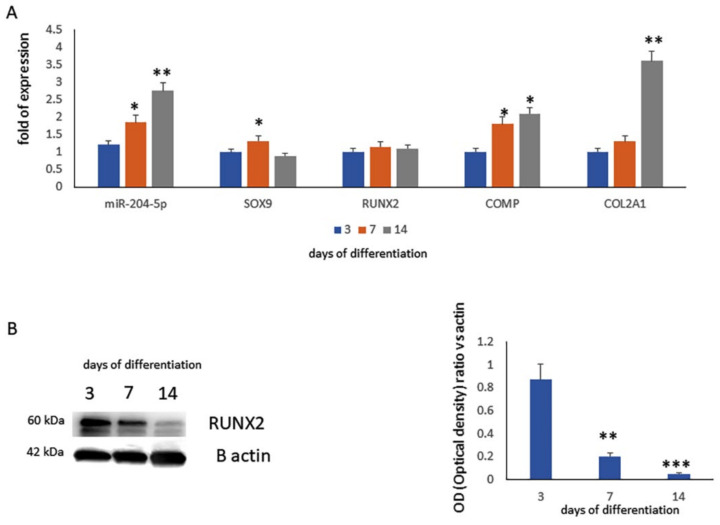
(**A**) MiR-204-5p and chondrogenic (SOX9, RUNX2, COMP, COL2A1) mRNA expression levels quantified by RT-PCR after 3, 7 and 14 days of chondrogenic differentiation in an MSCs in vitro model. RUNX2 protein expression levels assessed (**B**) by western blot analysis (left side) and quantified as a relative optical density ratio vs. B-actin (right side) on a protein lysate from an MSCs in vitro model after 3, 7 and 14 days of chondrogenic differentiation. (* *p* < 0.05; ** *p* < 0.005; *** *p* < 0.001 vs. expression levels of MSCs after 3 days of differentiation).

To evaluate the miR-204-5p modulation during chondrogenic differentiation, we performed in vitro experiments and analyzed the miR-204-5p expression as well as the expression of chondrogenesis-related genes in MSCs cultured in the presence of chondrogenic medium after 3, 7 and 14 days of differentiation. As shown in Figure 1A, miR-204-5p and the chondrogenic maturation-related genes’ expression increased upon differentiation. The expression of chondrogenic transcription factor SOX9 increased at the 7th day of differentiation. Since RUNX2 has been identified as a target of miR-204, we analyzed the gene expression and protein levels of RUNX2. While RUNX2 gene expression did not appear to be modulated, protein levels were greatly reduced during differentiation (Figure 1B). This suggests that miR-204-5p blocks RUNX2 at a post-transcriptional level.

**Figure 2:**
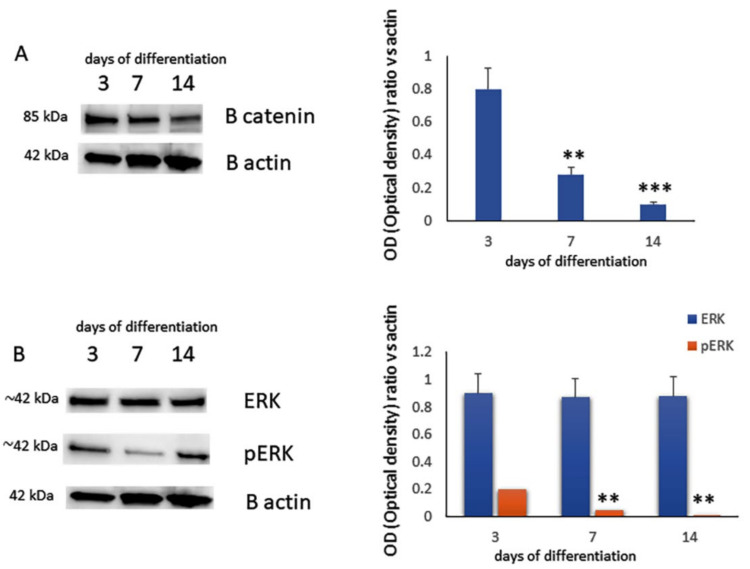
Protein levels assessed by western blot analysis (left side) and quantified as a relative optical density ratio vs. B-actin (right side) of (**A**) B-catenin and (**B**) ERK and pERK on protein lysates from MSCs after 3, 7 and 14 days of chondrogenic differentiation. (** *p* < 0.005; *** *p* < 0.001 vs. expression levels of MSCs after 3 days of differentiation).

As beta catenin plays an important role in chondrogenic differentiation, we analyzed the related protein levels during in vitro chondrogenic differentiation. We observed reduced beta catenin levels during chondrogenic differentiation (Figure 2A). Additionally, we also analyzed ERK and pERK levels, since it has been demonstrated that the extracellular signal-regulated kinase (ERK) is involved in chondrogenesis [23]. As shown in Figure 2B, reduced levels of pERK were observed during chondrogenesis (Figure 2B).

**Figure 3:**
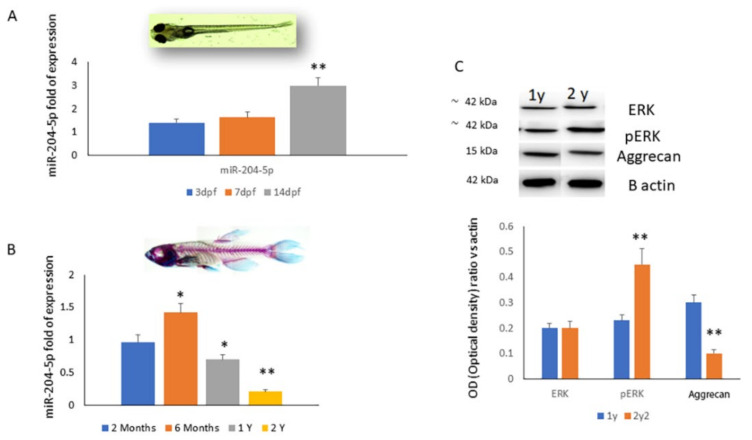
miR-204-5p mRNA expression levels quantified by RT-PCR during (**A**) embryogenesis (3, 7 and 14 days post fecundation (dpf)) and (**B**) aging (2 and 6 months, 1 and 2 years old) in a zebrafish larvae in vivo model. (**C**) ERK, pERK and aggrecan protein levels assessed by western blot (top) and quantified as a relative optical density ratio vs. B-actin (down) on protein lysates from a zebrafish larvae in vivo model. (* *p* < 0.05; ** *p* < 0.005 vs. expression levels of larvae after 3 (dpf) (**A**,**B**) and vs. 1 year old (**C**)).

To investigate the miR-204-5p modulation during the growth and aging processes, we performed in vivo analyses by using zebrafish larvae and an adult zebrafish model. We observed the upregulation of miR-204-5p expression in the larvae during embryogenesis evaluated at three, seven and 14 days post fertilization (dpf) (Figure 3A). Increased miR-204-5p expression was observed until six months of growth (Figure 3B). However, during aging, we observed a reduction of miR-204-5p expression (Figure 3B). The aging process was accompanied by a reduction of miR-204-5p expression, an increase in ERK phosphorylation and a decrease in aggrecan levels.

### 3.2. miR-204-5p Affects MSCs’ Commitment and Differentiation

**Figure 4:**
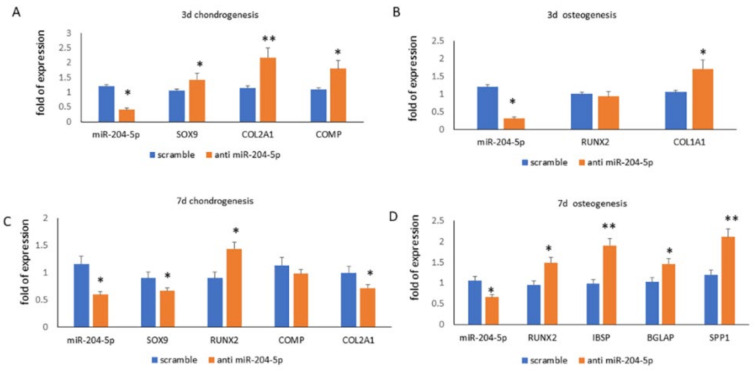
miR-204-5p, chondrogenic (SOX9, COL2A1, COMP, RUNX2) and osteogenic (RUNX2, COL1A1, IBSP, BGLAP, SPP1) mRNA expression levels quantified by RT-PCR during the early ((**A**,**B**) 3 days) and the middle ((**C**,**D**) 7 days) differentiation phases in miR-204-5p-silenced MSCs. (* *p* < 0.05; ** *p* < 0.005 vs. scramble/control).

After silencing miR-204-5p during the early differentiation phase, we observed the upregulation of SOX9 and chondrogenic related genes compared to controls. In particular, we observed that SOX9 and COL2A1 expression were 1.5- and 2.2-fold higher, respectively, compared to controls (Figure 4A). In addition, during osteogenesis, we observed the upregulation of COL1A1 (a RUNX2 downstream gene), whereas RUNX2 expression was slightly affected compared to controls (Figure 4B). Furthermore, the maturation of chondrocytes was considerably affected by miR-204-5p downregulation. In fact, in miR-204-5p-silenced cells, SOX9 and COL2A1 expressions were 0.67- and 0.7-fold, respectively (Figure 4C). On the contrary, positive effects of miR-204-5p silencing on osteogenic differentiation were also observed in the middle phase (Figure 4D).

**Figure 5:**
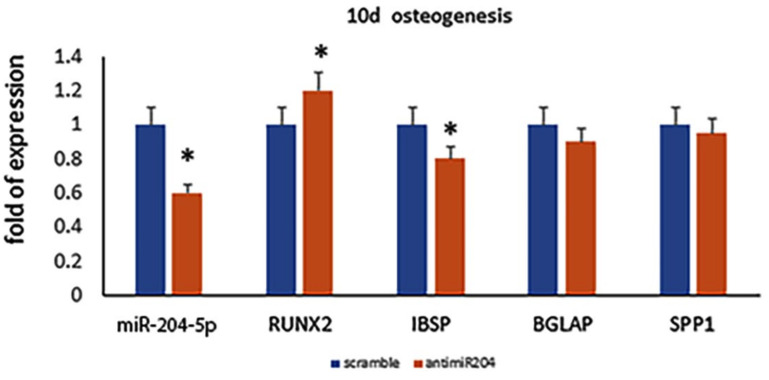
miR-204-5p and osteogenic (RUNX2, COL1A1, IBSP, BGLAP, SPP1) mRNA expression levels quantified by RT-PCR at a late (10 days) differentiation phase in miR-204-5p-silenced MSCs. (* *p* < 0.05 vs. scramble/control).

However, when we analyzed cells transfected with anti-miR-204-5p after 10 days of osteogenic differentiation, we observed a reduced expression of osteogenic maturation-associated genes (Figure 5).

**Figure 6:**
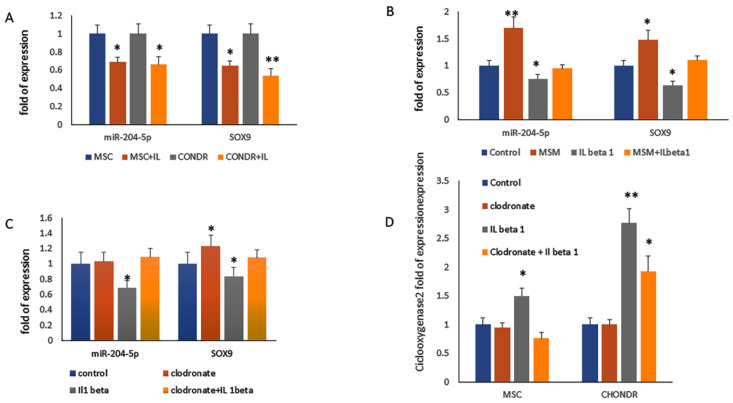
miR-204-5p and SOX9 mRNA expression levels quantified by RT-PCR in (**A**) MSCs during chondrogenesis (7 days post differentiation) and in chondrocytes after interleukin 1 beta (IL 1 beta), (**B**) methylsulphonylmethane (MSM) and (**C**) clodronate treatment. (**D**) Cyclooxygenase 2 mRNA expression levels quantified by RT-PCR in MSCs during chondrogenesis (7 days post differentiation) and in chondrocytes with and without IL 1 beta and clodronate treatment. (* *p* < 0.05; ** *p* < 0.005 vs. controls).

We analyzed the miR-204-5p modulation following inflammation stimuli such as those induced by interleukin 1 beta (IL 1 beta). As shown in Figure 6, we observed reduced levels of miR-204-5p as well as reduced levels of SOX9 in MSCs during chondrogenesis (after seven days of differentiation) and in chondrocytes treated with IL 1 beta (Figure 6A). Furthermore, we analyzed the effects of methylsulphonylmethane (MSM) antioxidant on miR-204-5p modulation in cells treated with IL 1 beta. As shown in Figure 6B, as a consequence of MSM addition, we observed an increase in miR-204-5p expression and a reversal of the effects of IL beta 1. We also analyzed the effects of clodronate, a non-aminobisphosphonate that is used in the management of osteoarthritis, on miR204-5p. We did not observe any effect of clodronate in the modulation of miR-204-5p in MSCs during chondrogenic differentiation (Figure 6C). However, clodronate was able to upregulate SOX9 expression, preventing miR-204 downregulation induced by IL beta 1 treatment (Figure 6C). Hence, to investigate the effects of clodronate in the prevention of IL beta1 effects, we analyzed the modulation of cyclooxygenase expression in MCSs during chondrogenesis and in chondrocytes with and without clodronate. As shown in Figure 6D, clodronate was able to prevent the upregulation of cyclooxygenase in IL beta 1 MSC cells and to reduce the levels of cyclooxygenase 2 in chondrocytes (Figure 6D).

### 3.3. miR-204-5p Affects Commitment and Differentiation of MSCs by Targeting RUNX2

**Figure 7:**
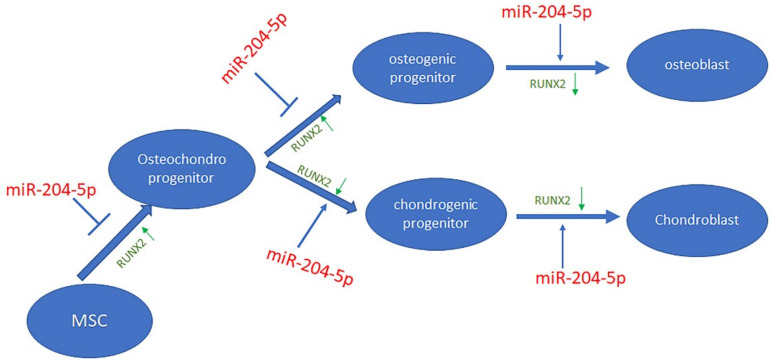
Mechanism of action of miR-204-5p during the commitment and the differentiation phases of MSCs towards chondrogenesis or osteogenesis. miR-204-5p reduces MSCs’ commitment to the osteochondrogenic lineage and promotes the maturation of osteoblasts and chondroblasts by modulating the RUNX2 expression. The expression levels of RUNX2 during differentiation are shown in green.

As shown in Figure 4 and Figure 5, RUNX2 gene expression levels were induced by anti -miR-204-5p treatment after seven and 10 days of differentiation. RUNX2 protein levels also increased in cells treated with anti-miR 204-5p, thereby confirming that miR-204 targets RUNX2, as previously reported. In fact, RUNX2 protein levels increased in MSCs cultured for seven days and treated with anti miR-204-5p (Figure S1). Accordingly, during chondrogenesis we observed that as miR 204-5p expression increased, RUNX2 gene expression levels did not change but protein levels decreased (Figure 1), suggesting a post-transcriptional regulation. Therefore, our data indicate that miR 204-5p may affect the osteochondrogenic commitment of MSCs and promote osteoblasts’ and chondroblasts’ maturation by modulating RUNX2 expression (Figure 7).

## 4. Discussion

Different signaling molecules induce the commitment and differentiation of MSCs by regulating the expression of transcription factors such as Runt-related transcription factor 2 (RUNX2), SRY-box 9 (SOX9) and Osterix (OSX) [8]. Epigenetic factors such as microRNAs play an important role in regulating bone and cartilage formation and homeostasis [24]. MicroRNAs are small noncoding RNAs that may act as negative regulators of the expression of specific mRNAs [8]. In addition, dysregulated miRNA expression is involved in skeletal disorders [10,25].

Various studies have demonstrated the involvement of miR-204 in MSCs’ fate and differentiation [17,19,26,27,28,29]. Most of these studies report that miR-204 inhibits osteogenic differentiation by targeting RUNX2. Moreover, contradictory data have been reported regarding its role in chondrocyte differentiation and in promoting or counteracting osteoarthritic pathology [18,30,31,32,33]. Therefore, with the aim of shedding light on the miR-204 role in chondrogenesis, we investigated its modulation during MSC differentiation and in response to growth. Our data demonstrated that miR-204-5p can either promote or reduce chondrogenesis by affecting specific phases of differentiation. During chondrogenesis, we observed an increased expression of miR-204-5p corresponding to the increased expression of chondrogenic transcription factor SOX9 and of chondrogenic maturation-associated genes such as COMP and COL2A. RUNX2 mRNA expression was not modulated during differentiation, but RUNX2 protein levels decreased during differentiation, confirming that miR-204 regulates RUNX2 post-transcriptionally [19]. During chondrogenic differentiation, we observed reduced levels of beta catenin, whose degradation is promoted by SOX9 [34], as well as reduced p-ERK levels, whose activation has been demonstrated to repress chondrocytic commitment [35]. Recently, it has been demonstrated that miR-204 blocks ERK pathway activation [36] and that the miR-204-5p/FOXC1/GDF7 axis regulates osteogenic differentiation [16]. Therefore, these findings, in combination with the inverse modulation between miR-204 and pERK observed in our study, suggest that miR-204 can affect the differentiation process by modulating important cellular signaling pathways. MiR-204 expression during development was investigated in larvae and adult zebrafish. Our data showed that miR-204-5p expression increased during embryogenesis and up to six months of growth. However, during aging we observed a reduced expression of miR-204-5p, as well as an increased ERK phosphorylation plus reduced levels of the maturation-associated marker aggrecan. In particular, it has been demonstrated that a decrease in ERK activity enhances chondrogenesis [37]. Therefore, our data showing increased pERK in aged zebrafish (>2 years old) suggest reduced chondrogenesis during the aging process. Interestingly, by silencing miR-204 during different phases of chondrogenic or osteogenic differentiation, we showed that miR-204 reduced MSCs’ osteochondrogenic commitment and increased osteoblasts’ and chondroblasts’ maturation. Based on our data, we believe that miR-204 regulates MSCs’ commitment to the osteochondrogenic lineage and the maturation of chondroblasts and osteoblasts by targeting the RUNX2 gene. In fact, it has been previously demonstrated that RUNX2 is expressed in osteochondroprecursors [38] and that its expression must be reduced to promote the maturation of osteoblasts [39] as well as the chondrogenic differentiation [40]. Finally, in order to test the miR-204 role in promoting or counteracting inflammation diseases, such as osteoarthritis, we treated chondrocytes or chondro-differentiating MSCs with IL beta 1. We observed a reduced expression of both miR-204-5p and SOX9 in IL beta 1-treated cells; the addition of the antioxidant methylsulphonylmethane reverted these effects. In addition, the treatment with clodronate, a non-aminobisphosphonate used to manage osteoarthritis, did not affect the modulation of miR-204-5p, but it was able to upregulate SOX9 expression and prevent miR-204-5p downregulation induced by Il beta 1 treatment. However, clodronate was able to prevent the upregulation of cyclooxygenase in cells treated with IL beta 1. These last findings suggest that clodronate may upregulate miR-204-5p indirectly by reducing inflammatory factors.

We believe that the data presented here may be useful in shedding light on the role of miR-204 in chondrocyte differentiation. In fact, previous studies reported either a positive [32] or a negative role [30] of miR-204 in chondrogenesis. Our study suggests that the negative or positive role of miR-204 depends on the phase of differentiation in which its expression increases. Furthermore, based on our data, we think that the role of miR-204 in differentiation most likely depends on its ability to reduce RUNX2 levels. Therefore, it would be interesting to investigate the miR-204 role in other pathways such as the ERK/c-Fos pathway or Smad signaling during the differentiation process.

## 5. Conclusions

In conclusion, our study demonstrates that miR-204-5p is highly modulated during chondrogenesis as well as during the aging process and that inflammatory stimuli may directly or indirectly affect its expression.

## Data Availability

The datasets used and/or analyzed during the current study are available from the corresponding author upon a reasonable request.

## Supplementary Materials

The following are available online at https://www.mdpi.com/article/10.3390/ijms23042130/s1
