# Supplementary material for: Modulation of miR-204 Expression during Chondrogenesis
Source: Int J Mol Sci. 2022 Feb 15;23(4):2130. doi: 10.3390/ijms23042130 (PMC8874780; doi:10.3390/ijms23042130)
Supplement: Supplementary file 1 [file ijms-23-02130-s001.zip › ijms-1568853-supplementary.pdf]

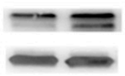

Figure S1: RUNX2 protein levels assessed by Western Blot in MSCs treated with (a) scramble or (b) anti miR-204-5p.
